# A Current Trend in Efficient Biopolymer Coatings for Edible Fruits to Enhance Shelf Life

**DOI:** 10.3390/polym16182639

**Published:** 2024-09-18

**Authors:** Ramkumar Vanaraj, Subburayan Manickavasagam Suresh Kumar, Gopiraman Mayakrishnan, Balamurugan Rathinam, Seong Cheol Kim

**Affiliations:** 1 Department of Computational Biology, Saveetha School of Engineering, Saveetha Institute of Medical and Technical Sciences, Saveetha University, Thandalam 602105, India; ramkumar@yu.ac.kr; 2 School of Chemical Engineering, Yeungnam University, Gyeongsan 38541, Republic of Korea; 3 Department of Chemistry, Dhanalakshmi Srinivasan Engineering College, Perambalur 621212, India; smsureshkumar2020@gmail.com; 4 Nano Fusion Technology Research Group, Institute for Fiber Engineering (IFES), Interdisciplinary Cluster for Cutting Edge Research (ICCER), Shinshu University, Tokida 3-15-1, Ueda 386-8567, Nagano, Japan; gopiraman@shinshu-u.ac.jp; 5 Department of Chemical and Materials Engineering, National Yunlin University of Science and Technology, 123 Univ. Rd., Sec. 3, Douliu 64002, Taiwan

**Keywords:** biopolymer, coatings, fruits, shelf life, sustainable, eco-friendly

## Abstract

In recent years, biopolymer coatings have emerged as an effective approach for extending the shelf life of edible fruits. The invention of biopolymer coverings has emerged as an innovation for extending fruit shelf life. Natural polymers, like chitosan, alginate, and pectin, are used to create these surfaces, which have several uses, including creating a barrier that prevents water evaporation, the spread of living microbes, and respiratory movement. These biopolymer coatings’ primary benefits are their environmental friendliness and lack of damage. This study highlights the advancements made in the creation and usage of biopolymer coatings, highlighting how well they preserve fruit quality, reduce post-harvest losses, and satisfy consumer demand for natural preservation methods. This study discusses the usefulness of the biopolymer coating in terms of preserving fruit quality, reducing waste, and extending the product’s shelf life. Biopolymer coatings’ potential as a sustainable solution for synthetic preservatives in the fruit sector is highlighted as are formulation process advances that combine natural ingredients and environmental implications. This essay focuses on the essential methods, such as new natural additives, as well as the environmental effect of biopolymer coatings, which are safe and healthy commercial alternatives.

## 1. Introduction

The preservation of food, particularly perishable items like fruits, has been a critical aspect of human survival and development [1,2,3,4,5]. Food preservation has always been a critical concern in the food industry. With the increasing demand for natural and sustainable methods to extend the shelf life of food products, biopolymer coatings have emerged as a promising solution. Derived from renewable sources, biopolymers offer an environmentally friendly alternative to synthetic materials [6,7,8,9,10]. This document explores the various biopolymers used in food coating applications, their benefits, and the latest advancements in this field. Traditional methods, such as drying, salting, and fermenting, have evolved over centuries, but the quest for innovative and efficient preservation techniques remains crucial in modern times. This need is driven by several factors, including the desire to reduce food waste, improve food security, and provide consumers with fresh produce that retains its nutritional and sensory qualities. One of the contemporary approaches to addressing these challenges is the development and application of biopolymer coatings for edible fruits [11,12,13,14,15]. Fruits are an essential component of the human diet, providing vital nutrients, antioxidants, and dietary fiber. However, their high water content and biological composition make them highly perishable. Post-harvest losses of fruits are a significant issue worldwide, with estimates suggesting that up to 50% of fruit production can be lost due to spoilage. This spoilage not only results in economic losses for producers and retailers but also contributes to food insecurity and environmental degradation. Traditional preservation methods, such as refrigeration, controlled atmosphere storage, and the use of chemical preservatives, have been widely used to extend the shelf life of fruits [16,17,18,19,20]. While these methods are effective to some extent, they have several limitations. Refrigeration and controlled atmosphere storage require significant energy inputs and infrastructure, making them less accessible in developing regions. Chemical preservatives, on the other hand, can have adverse effects on human health and the environment. Additionally, consumer preferences are shifting towards natural and minimally processed foods, creating a demand for alternative preservation techniques [21,22,23,24,25].

**Figure 1:**
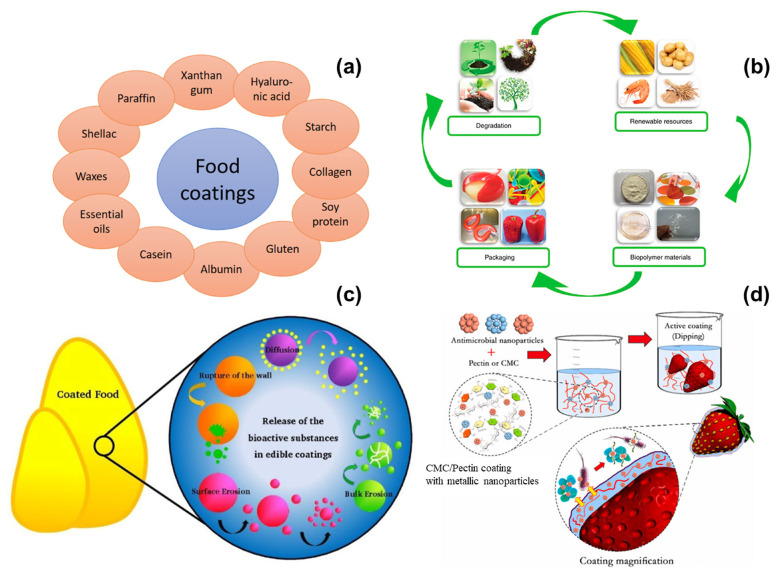
The different biopolymers used for food coating purposes (**a**) [51]; the source of sustainable materials for biopolymer (**b**) [52]; coating of biopolymer on the food materials and its surface micro-organisms. (**c**), [53]; the preparation of biopolymer nanocomposite and its processing mechanism on strawberries (**d**), the higher magnification on edible food surface (zoom in-part) [54].

The biopolymer coatings on the surface have emerged as a promising solution for the preservation of edible fruits. Biopolymers are natural polymers derived from renewable sources, such as plants, animals, and microorganisms [26,27,28,29]. They are biodegradable, are non-toxic, and often possess functional properties that can enhance the quality and shelf life of fruits. The application of biopolymer coatings involves covering the fruit with a thin layer of biopolymer material, which acts as a barrier to moisture, gases, and microbial contaminants [30,31,32,33]. This barrier can help to reduce respiration rates, delay ripening, and prevent spoilage. The biopolymer coatings offer several advantages over traditional preservation methods. They are environmentally friendly, as they are derived from renewable resources and are biodegradable. This aligns with the growing emphasis on sustainability and reducing the environmental impact of food production and preservation. Additionally, biopolymer coatings can be designed to be edible, eliminating the need for removal before consumption. This not only enhances convenience for consumers but also reduces food waste. Furthermore, biopolymer coatings can be customized to incorporate various functional additives, such as antimicrobial agents, antioxidants, and vitamins [34,35,36,37,38,39]. This can provide additional benefits, such as improved safety, nutritional value, and sensory qualities of the coated fruits. The biopolymer coating on food items has various advantages, including the following: sustainability: biopolymers are made from renewable resources and biodegrade, making them less hazardous to the environment than synthetic polymers; edibility and safety: many biopolymer coatings are safe, non-toxic, and edible, satisfying consumer demands for natural and clean-label goods; functional characteristics: biopolymer coatings can prolong shelf life and preserve food quality by having high barrier properties against moisture, oxygen, and microbiological contamination; customization: the properties of biopolymer coatings may be altered by modifying their composition and adding beneficial chemicals such as antioxidants, antimicrobials, and vitamins. Enhancing the shelf life and quality of food items may be achieved through the use of biopolymer coatings, which indicate great potential and sustainability. Biopolymer coatings hold great promise to transform food preservation, given the continuous progress in material science and application methodologies. To fully realize the promise of biopolymers in food coating applications, industry players must continue to innovate, conduct research, and work together. The versatility and potential for innovation in biopolymer coatings make them an exciting area of research and development in the field of food preservation. The development of biopolymer coatings for edible fruits is a dynamic and rapidly evolving field. Researchers and industry professionals are exploring various types of biopolymers, including polysaccharides, proteins, and lipids, to identify the most effective materials for specific fruit types and preservation goals [40,41,42,43,44,45]. Advances in nanotechnology and material science are also contributing to the development of novel biopolymer coatings with enhanced properties and functionalities. In addition to material innovations, there is a growing focus on optimizing the application methods and formulations of biopolymer coatings. Techniques such as dipping, spraying, and electrostatic coating are being investigated to ensure uniform coverage and adhesion of the coatings to the fruit surface. The incorporation of advanced analytical tools and techniques is also facilitating a better understanding of the interactions between biopolymer coatings and fruit surfaces, leading to more effective preservation strategies [46,47,48,49,50]. Figure 1a–d represent the biopolymer types and packing and preservation mechanism on the surface of food materials [51,52,53,54].

**Figure 2:**
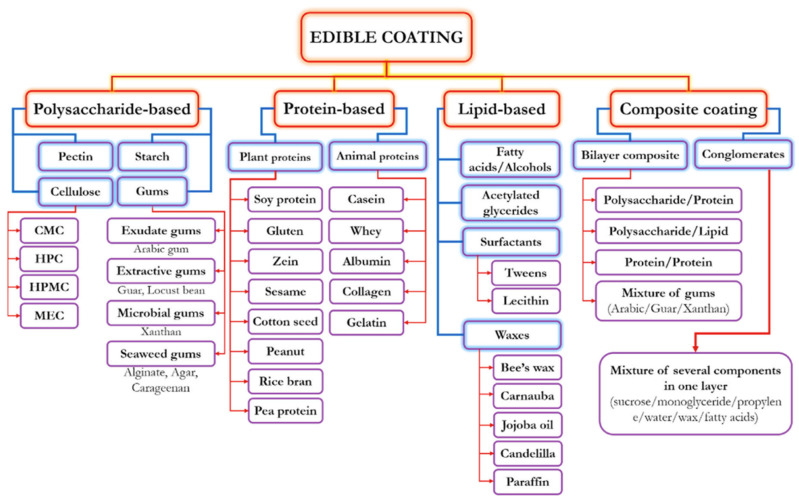
The types of edible coatings on food material and their subclasses [54].

There are a few types of biopolymers for food coating applications, and they are listed in Figure 2 [54]; the details are as follows. Polysaccharide biopolymers are large, naturally occurring compounds of long chains of monosaccharide units connected by glycoside linkages. They belong to the class of carbohydrates and are essential to many biological processes. The following are some salient features of polysaccharide biopolymers: chitosan: derived from chitin, found in the exoskeletons of crustaceans, chitosan has excellent film-forming properties and antimicrobial activity and is widely used for coating fruits, vegetables, and meat products [55,56,57]; starch: extracted from corn, potatoes, and other plants, starch-based coatings are biodegradable and can be modified to improve their mechanical properties and barrier functions [58,59,60]; cellulose and derivatives: cellulose, the primary component of plant cell walls, and its derivatives, like hydroxypropyl methylcellulose (HPMC), are used for their good film-forming ability and transparency [61,62,63,64]; proteins: proteins are significant, complex molecules that serve a variety of vital tasks in the body and are lengthy chains of amino acids necessary for properly constructing, operating, and controlling the body’s tissues and organs [65,66,67]. Here is a summary of the subclasses of proteins: whey protein is a byproduct of cheese production and is used for its excellent film-forming properties and ability to form barriers against oxygen and oil; soy protein is derived from soybeans, and soy protein coatings are known for their good barrier properties and biodegradability. Microorganisms can directly produce biopolymers from biomass or bioderived monomers, which are abundant and sustainable resources. The materials needed to create biopolymers are straightforward and reasonably priced; some of them are even present in agricultural waste. The mechanical and gas barrier properties of films and coatings can be improved by these proteins. Protein-based coatings can only restrict a limited amount of water since proteins are hydrophilic. Plasticizers and post-treatments can improve the performance of protein-based films and coatings. Protein-based films containing active compounds can effectively prevent or delay lipid oxidation and microorganism development. Gelatin is obtained from collagen and is used for its ability to form strong and flexible films, although it is sensitive to moisture [68,69,70]. Lipids are a broad class of hydrophobic organic compounds that serve important functions in the structure and function of living organisms. They are distinguished by being insoluble in water and soluble in nonpolar solvents [71,72,73]. Here are some classes of lipids: beeswax and carnauba wax: these natural waxes are used to create moisture-resistant coatings for fruits and vegetables, helping to reduce water loss and delay ripening; fatty acids: fatty acids and their derivatives can be used to create hydrophobic coatings that improve the water barrier properties of other biopolymers [74,75,76,77]. Composite materials are biopolymer composites formed by blending natural polymers (biopolymers) with other materials to improve mechanical, thermal, and physical characteristics [78,79,80,81]. Generally speaking, these composites are intended to be more eco-friendly than conventional composites manufactured from synthetic polymers. Some types of biopolymer composites are the following: polysaccharide–protein blends combine polysaccharides and proteins and can enhance the mechanical and barrier properties of the coatings; biopolymer–nanoparticle composites incorporate nanoparticles such as silver or zinc oxide and can provide antimicrobial properties and improve the overall functionality of the coatings.

Biopolymer coatings can have their mechanical strength, barrier qualities, and antibacterial activity improved by adding nanoparticles [82,83,84,85]. For example, silver nanoparticles can have a significant antibacterial impact, but clay nanoparticles can improve water vapor barrier qualities. Biopolymer coatings can be programmed to react to environmental changes or release active chemicals in response to specified stimuli. Coatings that, for example, release antimicrobial compounds in reaction to microbial development can offer an extra degree of defense. [86,87,88] Biopolymer coatings can be used to encapsulate bioactive substances, like probiotics, enzymes, and antioxidants, shielding them from degradation and transporting them to the intended location of action. Advancements in coating application processes, such as electrostatic spraying and layer-by-layer assembly, have resulted in more uniform and functional coatings [89,90,91,92,93,94,95]. Studies have shown that chitosan coatings can significantly extend the shelf life of strawberries by reducing microbial growth and delaying ripening [85,86,87,88]. Modified starch coatings have been used to reduce moisture loss and maintain the firmness of apples during storage. Whey protein coatings have been successfully applied to meat products to reduce lipid oxidation and microbial contamination, enhancing shelf life and safety. While biopolymers offer many advantages, the scalability and cost-effectiveness of their production and application remain challenges. Continued research and development are needed to optimize production processes and reduce costs. It is essential that the biopolymer coatings meet regulatory standards for food safety and quality for their widespread adoption [96,97,98,99,100].

This study aims to provide a comprehensive overview of current advances in efficient biopolymer coverings for edible fruits to extend shelf life. This work investigates the many classes of biopolymers that are being studied for fruit coverings, their functional properties, and the mechanisms by which they extend shelf life. This study also examines the most current advances in formulation and application procedures as well as the challenges and possible uses of this interesting preservation technology. A review of current research findings, case studies of effective applications, and opinions from professionals in the field are all included in this study’s scope. With this work, we hope to improve food security, reduce post-harvest losses, and advance sustainable food preservation methods by providing an in-depth understanding of the state of biopolymer coatings used in fruit preservation.

## 2. Cellulose-Based Biopolymers for Food Coating Applications

**Figure 3:**
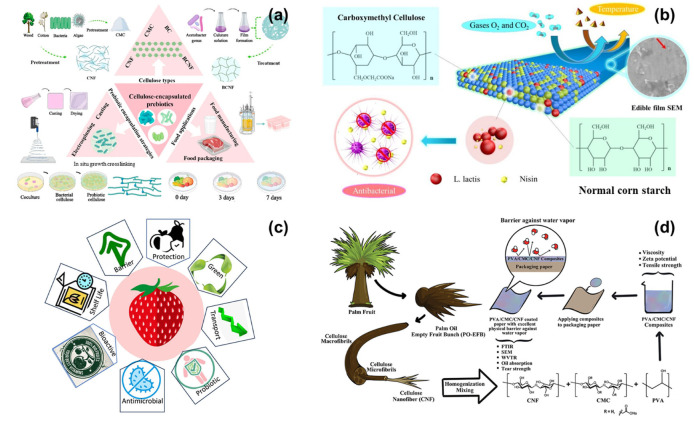
The cellulose-based biopolymers for edible food coatings, preparation and usages of cellulose (**a**) [102]; the antibacterial and protecting from the atmosphere mechanism of carboxymethyl cellulose (**b**) [102]; cellulose on strawberry fruit coatings and palm tree-derived cellulose for the coating applications (**c**,**d**) [103,104].

Cellulose, the most abundant natural polymer on earth, is a versatile and sustainable material widely used in the food industry. Derived from plant cell walls, cellulose and its derivatives have been extensively researched for their potential as biopolymer coatings for food applications (Figure 3a–d) [101,102,103,104]. This document explores the properties of cellulose-based biopolymers, their advantages, recent advancements, and case studies highlighting their effectiveness in food coating applications. Cellulose is non-toxic, biocompatible, and biodegradable, making it an excellent material for culinary applications while minimizing the environmental effect. Strong, flexible, and translucent films made of cellulose can act as efficient barriers to moisture and gasses, both of which are necessary to maintain the quality of food (Figure 3a). Cellulose and its derivatives have been generally recognized as safe (GRAS) by regulatory agencies, allowing for them to be utilized as edible coatings on a variety of food products. Films made of cellulose have high mechanical qualities and shield food goods from harm. Water-soluble methylcellulose creates efficient moisture-blocking films. It is frequently used to cover baked goods, fruits, and vegetables. Because of its superior ability to form films, hydroxypropyl methylcellulose is utilized to make flexible coatings that are resistant to fats and oils. Carboxymethyl cellulose (CMC) is a highly water-soluble derivative that forms transparent films. It is used in coatings to improve moisture retention and texture in food products [102,103].

Nanocellulose, including cellulose nanocrystals (CNC) and cellulose nanofibrils (CNF), has unique mechanical properties and can enhance the strength and barrier properties of coatings. CMC, a flexible cellulose derivative generated from a variety of lignocellulosic sources, is gaining popularity as an edible food covering. In this study, the synthesis of CMC from empty fruit bunches (EFB) is evaluated as a potential edible food coating material using a systematic review method. It looks into several eco-friendly CMC production processes including green cellulose pretreatments. The evaluation includes a discussion of formulation procedures; coating quality and safety; and commercial feasibility in comparison to other materials and CMC-based coatings (Figure 3b). Food coating and CMC are related by bibliometric study. Consequently, this study found that research on edible food coatings made from CMC for a variety of applications in the food sector has significantly increased. Cellulose coatings can significantly extend the shelf life of food products by reducing moisture loss, slowing down respiration rates, and preventing microbial growth. These coatings help maintain the sensory and nutritional quality of food by providing effective barriers against oxygen and other gases. Being natural and biodegradable, cellulose-based coatings do not pose the health risks associated with synthetic preservatives and reduce environmental pollution from packaging waste. Cellulose-based coatings can be tailored to meet specific requirements by modifying their composition and incorporating functional additives, such as antioxidants, antimicrobials, and colorants. Incorporating nanocellulose into the coatings can improve their mechanical strength, transparency, and barrier properties. Nanocomposite coatings can provide superior protection against moisture and gases. Adding natural extracts, essential oils, and bioactive compounds to cellulose-based coatings can enhance their antimicrobial and antioxidant properties, providing additional benefits for food preservation [103].

Techniques such as electrospinning and layer-by-layer assembly are being explored to create more uniform and effective coatings with improved functionality. Research is focused on developing more sustainable methods for producing cellulose and its derivatives, including using agricultural waste and other renewable resources [101]. Fruits and vegetables: Coating apples with HPMC has been demonstrated to minimize weight loss, retain firmness, and increase shelf life during storage. Cellulose-based coverings can help keep cucumbers fresh and visually appealing by decreasing dehydration and microbiological deterioration. Non-vegetable products: Applying CMC coatings to chicken fillets can increase moisture retention and lower microbiological contamination, maintaining quality and prolonging shelf life [102,103]. Applying cellulose coatings to fish can help prevent oxidation and rotting, ensuring their freshness and safety. Milk product: Cheese coated with cellulose-based materials can stop mold development and moisture loss, preserving the product’s quality and prolonging its shelf life. Edible films manufactured from cellulose derivatives can be utilized to make biodegradable, single-serving yogurt packaging that extends the product’s shelf life. Baked goods: Bread can have its shelf life extended while retaining its texture and flavor by using methylcellulose coatings to stop staling and mold development. Cakes coated with HPMC will retain their freshness and suppleness over time and lose less moisture. Although cellulose-based coatings have several advantages, in order to make them more commercially feasible for general usage, issues with manufacturing and application cost and scalability must be resolved [104,105]. Cellulose-based biopolymers offer a sustainable and effective solution for food coating applications. Their natural abundance, biocompatibility, and excellent film-forming properties make them ideal for extending the shelf life and preserving the quality of various food products. With ongoing advancements in material science and application techniques, the potential for cellulose-based coatings to revolutionize food preservation is significant. Continued research and innovation will be key to unlocking the full potential of cellulose in the food industry.

## 3. Starch-Based Biopolymers for Food Coating Applications

Starch, a natural polysaccharide derived from various plant sources such as corn, potatoes, rice, and wheat, has garnered significant attention as a sustainable material for food coating applications [106]. Its biocompatibility, film-forming properties, and abundance make it an excellent candidate for extending the shelf life and preserving the quality of food products. This document explores the properties of starch-based biopolymers, their advantages, recent advancements, and case studies showcasing their effectiveness in food coating applications. Starch is non-toxic, biocompatible, and biodegradable, making it safe for food applications and environmentally friendly. Starch can form strong, transparent, and flexible films that serve as effective barriers to gases and moisture, essential for preserving food quality. Starch-based films have good mechanical strength and can be modified to improve their flexibility and durability. Extracted directly from plants, native starch can form films but often requires modification to enhance its properties for specific applications [106]. Chemical, physical, or enzymatic modifications can improve the functional properties of starch, such as its solubility, film-forming ability, and mechanical strength [107,108]. Combining starch with other biopolymers, such as proteins or lipids, can enhance the performance of the resulting coatings by improving their barrier and mechanical properties. Starch coatings can significantly extend the shelf life of food products by reducing moisture loss, slowing down respiration rates, and preventing microbial growth. These coatings help maintain the sensory and nutritional quality of food by providing effective barriers against oxygen and other gases. Being natural and biodegradable, starch-based coatings do not pose the health risks associated with synthetic preservatives and reduce environmental pollution from packaging waste. Starch-based coatings can be tailored to meet specific requirements by modifying their composition and incorporating functional additives, such as antioxidants, antimicrobials, and colorants [109,110].

The mechanical strength, transparency, and barrier qualities of starch coatings can all be enhanced by adding nanoparticles, such as clay, silver, or zinc oxide. Coatings with nanocomposite materials offer better defense against gasses and moisture. To improve the antibacterial and antioxidant qualities of starch-based coatings and improve food preservation, natural extracts, essential oils, and bioactive substances can be added [107]. Methods like layer-by-layer assembly and electrospinning are being investigated to produce more functional coatings that are more consistent and efficient. Research is focused on creating starch-based coatings using more environmentally friendly processes, such as using agricultural waste and other renewable resources [108,109,110]. It has been demonstrated that starch-based coatings prevent weight loss, preserve texture, and increase the shelf life of stored goods. Starch coatings can help maintain the freshness and visual appeal of strawberries by reducing dehydration and microbial spoilage [108,109]. Starch coatings on non-vegetable foods can minimize microbial contamination and enhance moisture retention, increasing shelf life and preserving quality. Starch coatings on seafood can help prevent oxidation and spoiling, ensuring freshness and safety. A starch solution used as a coating on cheese can stop mold from growing and moisture from evaporating, preserving the product’s quality and prolonging its shelf life. Edible films derived from starch can be utilized to make biodegradable, single-serving curd packets that extend the product’s shelf life [110]. The bakery food can have its shelf life extended while retaining its texture and flavor with the use of starch coatings, which can help stop staling and mold development. Foods that have starch on them can retain their freshness and suppleness over time and lose less moisture. While starch-based coatings offer many benefits, the cost and scalability of production and application need to be addressed to make them more economically viable for widespread use.

**Figure 4:**
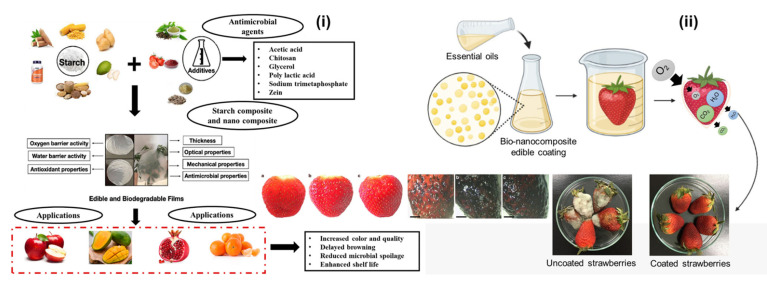
The starch-based biopolymers for the different food coating purposes (**i**) [106]; the real time usage of starch coating on edible strawberry fruit on various days (**ii**) [107]; *insert*: (**a**–**c**) the strawberry with and without coatings of biopolymer [108].

Because starch-based films include antibacterial, antioxidant, UV resistance, oxygen and moisture barrier, and pH-reactive qualities, they can increase the shelf life of foods. The shortcomings of pure starch-based materials, such as their susceptibility to moisture and the lower tensile capabilities of natural polymers, have led to the development of several composite materials (Figure 4(i)). The production of completely biodegradable starch-based polymers is impacted by the addition of any additives. The biggest hurdle to the commercialization of biodegradable packaging film generated from starch is structural variations in starch complex molecules, which have a considerable impact on the films’ functional qualities. Starch-based biopolymers offer a sustainable and effective solution for food coating applications (Figure 4(ii),a–c). Their natural abundance, biocompatibility, and excellent film-forming properties make them ideal for extending the shelf life and preserving the quality of various food products. With ongoing advancements in material science and application techniques, the potential for starch-based coatings to revolutionize food preservation is significant. Continued research and innovation will be key to unlocking the full potential of starch in the food industry.

## 4. Chitosan-Based Biopolymers for Food Coating Applications

**Figure 5:**
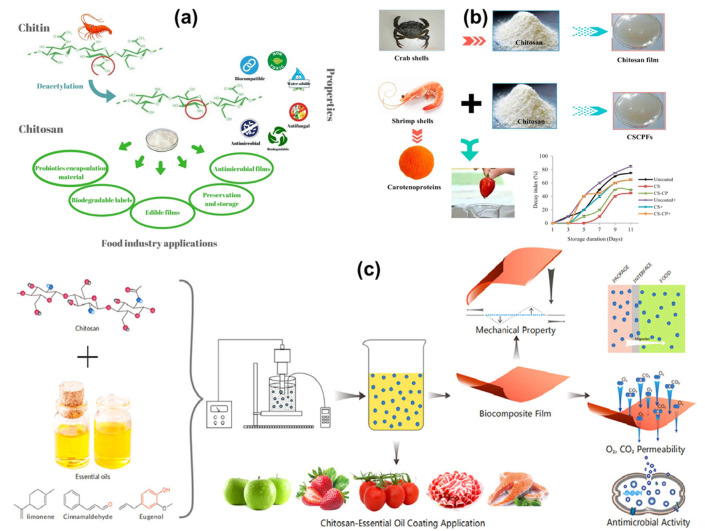
The chitosan derived from chitin and its multipurpose applications (**a**) [111]; the preparation of chitosan from shrimp and crab for edible strawberry coating applications (**b**) [112]; the preparation of food coating solution by chitosan and bio-solvent for multi-food coating applications (**c**) [113].

Chitosan, a biopolymer formed from chitin found in crab and insect exoskeletons, is gaining popularity in the food sector due to its promise as a natural and sustainable food coating ingredient [111,112,113]. Chitosan is biocompatible and biodegradable, safe to use in food applications, and beneficial to the environment [111]. It has intrinsic antibacterial qualities against a wide range of bacteria, fungi, and yeasts, which serve to increase the shelf life of food goods. In order to effectively block gasses and moisture—two elements that are essential for maintaining the quality of food—chitosan can make translucent, flexible films (Figure 5a–c). Because regulatory organizations typically see chitosan as safe, it can be employed as an edible coating on a variety of food products. Chitosan, a biopolymer generated from chitin, has attracted substantial attention in the food industry due to its unique qualities as an edible coating ingredient. It is a great option to improve food quality and shelf life because of its biocompatibility, non-toxicity, and biodegradability. Chitosan’s antibacterial and antifungal capabilities aid in suppressing the growth of infections and spoilage germs, which is critical for preserving the freshness of perishable items. A useful moisture and gas barrier are also provided by chitosan’s superior film-forming capacity, which slows down the pace at which food oxidizes and dehydrates. This helps to postpone spoiling while maintaining texture and taste [112]. Furthermore, chitosan coatings can be combined with functional elements, such as antioxidants, minerals, and taste enhancers, to boost the nutritional value and attractiveness of food items. Its compatibility with various biopolymers as well as its flexibility in producing composite films improve its suitability for food use. Chitosan’s unique mix of antibacterial activity, film-forming capabilities, and flexibility makes it a viable material for the creation of sustainable and practical food-packaging solutions [112,113]. Chitosan can be combined with other biopolymers and functional additives to enhance its properties and tailor it for specific food applications.

The antibacterial and barrier qualities of chitosan coatings can be improved by adding nanoparticles, such as silver, zinc oxide, or titanium dioxide [113]. Adding natural antioxidants (e.g., essential oils) or vitamins to chitosan coatings can boost health and enhance food preservation. Chemical changes, such as grafting or cross-linking, can increase chitosan’s mechanical strength, solubility, and film-forming capabilities. Chitosan coatings work better when applied using advanced techniques like layer-by-layer deposition and electrostatic spraying, which guarantee consistent adherence and coating [113]. Chitosan coatings have been shown to extend the shelf life of strawberries by inhibiting fungal growth and reducing respiration rates [114]. Coating tomatoes with chitosan helps maintain firmness, color, and nutritional quality during storage. Chitosan coatings on chicken and fish fillets can reduce microbial contamination and lipid oxidation, preserving freshness and safety. Applying chitosan coatings on shrimp helps prevent microbial growth and extend shelf life during refrigerated storage. Chitosan coatings on cheese can inhibit mold growth and prevent moisture loss, maintaining the quality of the product over extended periods. Chitosan can be used as a coating to extend the shelf life of bread by preventing mold growth and retaining moisture [115,116].

Chitosan-based biopolymers offer a promising and sustainable solution for food coating applications. Their natural antimicrobial properties, biodegradability, and ability to form effective barrier films make them ideal for extending the shelf life and preserving the quality of various food products. With ongoing advancements and research, chitosan coatings have the potential to revolutionize food preservation and contribute to a more sustainable food system.

## 5. Gelatin-Based Biopolymers for Food Coating Applications

Gelatin, a natural protein derived from collagen found in animal connective tissues, bones, and skin, has gained significant attention in the food industry as a versatile and sustainable material for food coating applications [116,117,118]. Its unique film-forming properties, biocompatibility, and biodegradability make it an excellent candidate for extending the shelf life and preserving the quality of food products. This document explores the properties of gelatin-based biopolymers, their advantages, recent advancements, and case studies showcasing their effectiveness in food coating applications. Gelatin is acceptable for use in food applications and ecologically benign since it is non-toxic, biocompatible, and biodegradable. Strong, pliable, and translucent films made of gelatin can effectively block gasses and moisture, two things that are necessary to maintain the quality of food. Gelatin coatings can dramatically increase the shelf life of food goods by minimizing moisture loss, lowering respiration rates, and inhibiting microbiological development [117]. As efficient barriers against oxygen and other gases, these coatings aid in the preservation of the food’s sensory and nutritional qualities. Gelatin-based coatings are natural and biodegradable, which means they do not have the health concerns of synthetic preservatives. Gelatin coatings can help prevent staling and mold growth in bread, extending its shelf life while maintaining texture and flavor. Coating cakes with gelatin can reduce moisture loss and maintain their softness and freshness over time. They also lessen environmental pollution from packaging waste [118]. Gelatin-based coatings can be tailored to meet specific requirements by modifying their composition and incorporating functional additives, such as antioxidants, antimicrobials, and colorants [119]. Nanocomposite coatings provide superior protection against moisture and gases.

Strawberries with gelatin coatings have been demonstrated to have longer shelf lives due to the inhibition of microbiological development and preservation of firmness and color. Gelatin coatings help keep apples fresher longer in storage by reducing oxidation and preventing moisture loss [120,121]. On protein-rich, non-vegetarian food, gelatin coatings can improve moisture retention and lower microbiological contamination, extending shelf life and preserving quality. Seafoods may be kept fresh and safe by using gelatin coatings to stop oxidation and spoiling. A gelatin coating on milk products can stop mold from growing and moisture from evaporating, preserving the product’s quality and prolonging its shelf life. The curd single-serving containers can be manufactured with edible films derived from gelatin derivatives, which will extend the shelf life of the food and make it biodegradable [122]. Gelatin is a biopolymer with great promise for use as a coating because of its exceptional functional and technical properties, affordability, and ease of use. Antioxidants and other bioactive substances can extend the shelf life of extremely perishable items by delaying oxidation.

The gelatin-based coatings offer many benefits; however, the cost and scalability of production and application need to be addressed to make them more economically viable for widespread use. Because of its biocompatibility and biodegradability, gelatin is a safe and sustainable material for edible coatings. To further increase food quality and safety, active substances, including taste enhancers, antioxidants, and antimicrobials, may be easily included in gelatin due to its great degree of adaptability. Because of the material’s excellent solubility, gelation characteristics, and compatibility with other biopolymers, coatings may be customized to fit a variety of products, such as fruits, vegetables, meats, and confections. Furthermore, gelatin coatings can retain or improve the sensory characteristics of food, keeping texture and appearance while reducing spoilage [122]. A superior gelatin coating enhanced weight loss and changed the color of the meat, among other physicochemical changes; nonetheless, the meat’s pH and water activity remained constant while being preserved, preventing meat degradation. Moreover, the thicker coating prevented the meat’s lipids from oxidizing, and the cooked meat exhibited a high degree of antioxidant activity [120,121,122].

**Figure 6:**
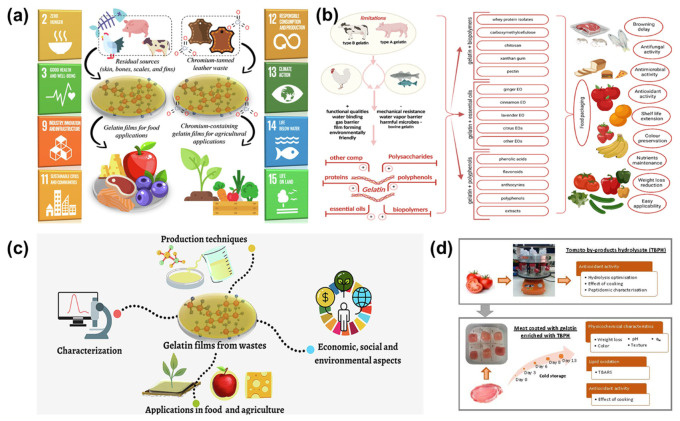
The gelatin obtained from residual sources for food coating purposes (**a**) [118]; the preparation of gelatin-based solutions with different solvents for food coatings (**b**) [117]; the gelatin materials applied in food and agriculture areas (**c**) [118]; the real-time usage of gelatin for meat coating bio application (**d**) [119].

Gelatin-based biopolymers offer a sustainable and effective solution for food coating applications (Figure 6a–d). Their natural abundance, biocompatibility, and excellent film-forming properties make them ideal for extending the shelf life and preserving the quality of various food products. With ongoing advancements in material science and application techniques, the potential for gelatin-based coatings to revolutionize food preservation is significant. Continued research and innovation will be key to unlocking the full potential of gelatin in the food industry.

## 6. Fatty Acid and Wax Coatings for Extending Shelf Life of Food Materials

**Figure 7:**
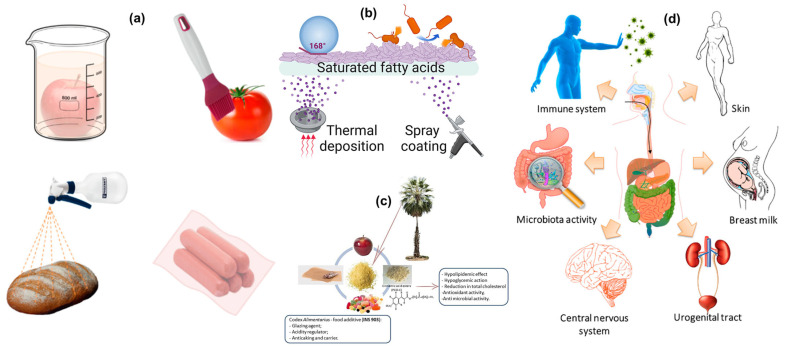
The different types of fatty acid coatings (dipping, brush, spray, packing) food materials (**a**) [123]; surface mechanism of the fatty acid from bacterial infection (**b**) [124]; the palm tree-derived fatty acid for the food coating needs (**c**) [125]; the advantages on probiotic-based food coating material intake on several human health benefits (**d**) [126].

Fatty acids and waxes are natural substances that have been extensively used as coatings to extend the shelf life of food materials [123,124,125]. Lipids are a class of chemicals that includes fats. Triglycerides, which are composed of one glycerol and three fatty acids, are the primary components of fats and other lipids. These coatings form protective barriers on the surface of foods, reducing moisture loss, gas exchange, and microbial contamination, thereby preserving freshness and quality. This document explores the properties of fatty acid and wax coatings, their advantages, recent advancements, and case studies showcasing their effectiveness in food preservation (Figure 7a–d). Both fatty acids and waxes are hydrophobic, meaning they repel water. This property helps in creating an effective moisture barrier on the surface of food products. Fatty acids and waxes can form continuous, flexible films that adhere well to the surface of food products, providing a protective layer. These substances are naturally occurring and biodegradable, making them environmentally friendly options for food coatings [123]. Many fatty acids and waxes are considered safe for consumption and are approved for use as food additives by regulatory bodies, such as the FDA and EFSA. Oleic acid: Commonly found in olive oil and used to create moisture-resistant coatings. Stearic acid: Derived from animal fats and cocoa butter, used for its film-forming properties and stability. Beeswax: Produced by honeybees, beeswax is widely used in fruit coatings to reduce moisture loss and delay ripening. Carnauba wax: Obtained from the leaves of the Brazilian palm tree, it is used for its excellent barrier properties and glossy finish. Candelilla wax: Extracted from the leaves of the candelilla shrub, used similarly to carnauba wax for its protective and aesthetic properties. Paraffin wax: Derived from petroleum, paraffin wax is used for coating cheese and other food products to prevent moisture loss and microbial contamination. These coatings can significantly extend the shelf life of food products by reducing moisture loss, controlling gas exchange, and inhibiting microbial growth [124]. Advanced application methods, such as electrostatic spraying and layer-by-layer assembly, ensure uniform coating and adhesion, enhancing the effectiveness of fatty acid and wax coatings. Research is focused on developing sustainable methods for sourcing fatty acids and waxes, including utilizing agricultural byproducts and renewable resources [125,126,127]. Apples: Beeswax and carnauba wax coatings have been shown to reduce weight loss, maintain firmness, and extend the shelf life of apples during storage. Citrus fruits: Wax coatings help maintain the freshness and visual appeal of citrus fruits by reducing dehydration and microbial spoilage. Fatty acid coatings on chicken-based products can reduce microbial contamination and improve moisture retention, extending shelf life and maintaining quality [127]. Fatty acid and wax coatings contribute to the preservation of food products’ nutritional value and sensory appeal by creating a barrier of defense. Many fatty acids and waxes are naturally occurring and biodegradable, and thus, they do not present the same health dangers as synthetic preservatives. They also help to mitigate the environmental pollution caused by packaging waste. A vast variety of food items, such as fruits, vegetables, meat, fish, dairy, and baked goods, can be coated with these coatings. Nanoparticles can improve the mechanical strength, barrier qualities, and antibacterial activity of fatty acid and wax coatings. Nanocomposite coatings offer the best defense against gasses and moisture. Enhancing the antibacterial and antioxidant capabilities of these coatings with the addition of natural extracts, essential oils, and bioactive substances can yield further benefits for food preservation [125]. Wax coatings for fish can prevent oxidation and rotting, ensuring freshness and safety. Milk products with paraffin wax coatings can keep their quality and prolong their shelf life by preventing moisture loss and mold growth. Bread may have its shelf life extended while retaining its texture and flavor by using fatty acid coatings to help stop staling and mold development. The bakery products coated in wax will hold their freshness and suppleness longer and loose less moisture.

The characteristics and morphology of fatty acids that self-assemble into highly hierarchical crystalline structures with a water contact angle of approximately 165 and a contact angle hysteresis of less than six depend on the individual fatty acid utilized and the deposition process. Fatty acid coatings also have exceptional heat resilience. This novel coating family effectively prevents biofouling and bacterial growth in Gram-positive and Gram-negative bacteria (Escherichia coli and Listeria innocua, respectively). These versatile coatings offer significant potential for usage in a range of industries, including biomedicine and food safety, because they provide safe and sustainable solutions. Wax and fatty acid coatings provide a viable and efficient way to increase food goods’ shelf lives while maintaining their quality. They are perfect for many food coating applications because of their hydrophobic qualities, biodegradability, and natural abundance. With continued advances in material science and application techniques, the potential for these coatings to transform food preservation is enormous. Realizing the complete potential of fatty acids and waxes in the food sector will need ongoing research and innovation [126,127].

## 7. Biopolymer Nanocomposite Materials for Food Coating Applications

**Figure 8:**
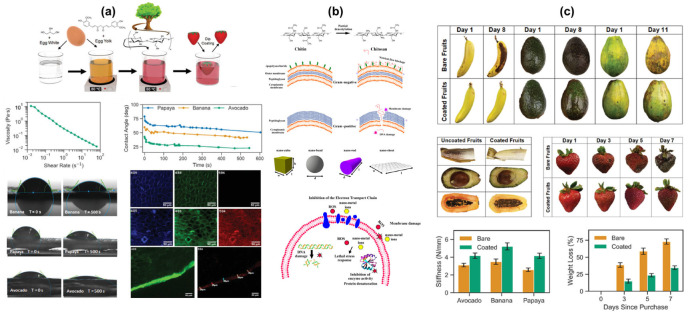
The biopolymer and its composites for the food coating applications (**a**–**c**); the biopolymer composites on papaya, banana, and avocados, with water contact angle and microscopic images (**a**) [128]; the surface mechanism of chitosan nanocomposites for the food coating applications (**b**) [129]; the real-time images of banana, avocado, papaya, and strawberry fruits on biopolymer nanocomposite coatings (**c**) [128].

The food industry is continually seeking innovative methods to extend the shelf life and maintain the quality of food products [128,129,130]. One such promising approach is the use of biopolymer nanocomposite materials for food coatings. These materials combine the natural, biodegradable properties of biopolymers with the enhanced functional properties conferred by nanoparticles (Figure 8a–c) [128]. Biopolymers’ ability to withstand moisture, gasses, and microbiological pollutants is enhanced when they contain nanoparticles. The mechanical strength and flexibility of biopolymer films may be greatly increased by nanoparticles, increasing their durability and damage resistance. Certain nanoparticles, such as silver and zinc oxide, have antibacterial capabilities that can help reduce the growth of spoilage and harmful microorganisms on food surfaces. Because biopolymer nanocomposites preserve the biocompatibility and biodegradability of their parent biopolymers, they are suitable for use in food applications and have minimal environmental impact [129]. Chitosan: For enhanced usefulness, chitosan is frequently coupled with nanoparticles. It is well known for its superior film-forming abilities and intrinsic antibacterial activity. Starch: Easily obtainable and biodegradable, starch can create robust films and is frequently combined with different types of nanoparticles [130,131,132]. Cellulose and its derivatives: Cellulose nanocrystals (CNC) and nanofibrils (CNF) are particularly effective in improving the mechanical and barrier properties of biopolymer films. With excellent film-forming abilities and good barrier properties, whey protein is used in nanocomposite coatings for various food products. Derived from soybeans, soy protein films benefit from the addition of nanoparticles to enhance their properties. These natural waxes are often used in combination with biopolymers to improve the water resistance and flexibility of the films. Silver nanoparticles (AgNPs) are known for their strong antimicrobial properties; AgNPs can significantly enhance the shelf life of coated food products. Zinc oxide nanoparticles (ZnO NPs) provide antimicrobial activity and UV protection, making them ideal for food coatings. Titanium dioxide nanoparticles (TiO_2_ NPs) offer antimicrobial properties and improve the mechanical strength of biopolymer films. Montmorillonite (MMT) clay mineral improves the barrier properties of biopolymer films against gases and moisture. Halloysite nanotubes (HNTs) enhance the mechanical strength and barrier properties of biopolymer coatings. Carbon nanotubes (CNTs) can significantly enhance the mechanical strength and electrical properties of biopolymer films. Graphene oxide (GO) improves the barrier properties and mechanical strength of biopolymer coatings. The enhanced barrier and antimicrobial properties of biopolymer nanocomposite coatings can significantly extend the shelf life of food products. These coatings help maintain the sensory and nutritional quality of food by providing effective barriers against moisture, oxygen, and microbial contamination. Biopolymer nanocomposites are generally safe for consumption and environmentally friendly, reducing the need for synthetic preservatives and packaging materials. Biopolymer nanocomposite coatings can be tailored to meet specific requirements by selecting appropriate biopolymer and nanoparticle combinations and functional additives. Biopolymer nanocomposites are being developed with responsive properties, such as releasing antimicrobial agents in response to microbial growth or changes in temperature and humidity. Research is focused on using renewable resources and green synthesis methods to produce nanoparticles and biopolymer nanocomposites, reducing the environmental impact [133]. Electrospinning, solvent casting, and layer-by-layer assembly are all being refined to provide consistent and effective biopolymer nanocomposite coatings [134,135], combining various nanoparticles with biopolymers to produce multifunctional coatings that have several advantages, such as mechanical strength, UV protection, and antibacterial activity. Research has demonstrated that chitosan–AgNP nanocomposite coatings can inhibit microbial development and prolong the shelf life of strawberries without compromising their taste attributes. Starch-based nanocomposite coatings containing ZnO NPs aid in preserving firmness, color, and nutritional value throughout storage. Whey protein–AgNP nanocomposite coatings can reduce microbial contamination while improving moisture retention, hence increasing shelf life and maintaining quality [136]. Applying gelatin-based nanocomposite coatings with TiO_2_ NPs to fish can help prevent oxidation and spoilage, preserving freshness and safety. Cellulose nanocrystal (CNC) nanocomposite coatings on cheese can inhibit mold growth and prevent moisture loss, maintaining the quality and extending the shelf life of the product. Starch–CNT nanocomposite coatings can help prevent staling and mold growth in bread, extending its shelf life while maintaining texture and flavor. Coating cakes with protein-based nanocomposites can reduce moisture loss and maintain their softness and freshness over time.

A viable and environmentally friendly option for food coating applications is provided by biopolymer nanocomposites. They are perfect for increasing the shelf life and maintaining the quality of different food goods because of their improved barrier, mechanical, and antibacterial qualities [134,135]. With continued breakthroughs in material science and application methodologies, biopolymer nanocomposites can revolutionize food preservation. Leveraging the full potential of these materials in the food business will need persistent research and innovation [136,137].

## 8. Removal of Biopolymers from Food Materials and Quantitative Features of Biopolymer Coating

Food coating materials may be removed before consumption to ensure cleanliness and safety. Some examples of coating materials that might be contaminated with chemicals and possibly dangerous bacteria are fruit wax, leftover insecticides, and packaging residues. It is advised to give the food a good wash under running water to remove these coatings and to use a brush on rougher surfaces like potatoes or apples. Rinsing after soaking in water may be quite helpful for leafy greens. One of the natural cleaning options that could assist in eliminating undesirable residues is diluted vinegar. In addition to improving the food’s flavor and texture, eliminating all coating substances also enhances the customer’s safety and health [138,139,140].

The quantitative aspects of the effect of food coating materials include measuring and analyzing numerous factors that affect food preservation, quality, and shelf life [141,142,143]. Food coatings are used to keep food fresher longer, make it look better, and keep bacteria out of it. Quantitative analysis is often used to evaluate coating qualities such as thickness, permeability, and mechanical strength [144,145,146]. Moisture retention, oxygen transfer rate (OTR), and barrier qualities are important because they keep texture and stop spoiling. Studies also evaluate the effect on sensory characteristics, such as texture, color, and taste. The effectiveness of the coatings under various storage situations is frequently predicted using mathematical models [147,148]. Quantitative evaluations optimize coating formulations for improved food safety and quality. Quantitative elements of food coating materials might include assessing how much the coating improves food storability and quality when compared to uncoated alternatives. Coatings can extend the shelf life of foods by two to three times, depending on the type of coating and the food product. Edible coatings, such as chitosan or alginate, have been proven to reduce moisture loss, delay ripening, and limit microbial formation, resulting in a significant gain in storage capacity. Comparing the rate of spoilage, weight loss, and nutritional deterioration of coated versus uncoated goods over time is a standard way to assess this impact. Additionally, by strengthening the product’s resistance to oxidation and outside factors, these coatings may enhance preservation and prolong freshness [149,150]. The recent trends on bioactive materials and polymers on food coating applications are listed in Table 1.

## 9. The Structural Properties Relationship of Biopolymers for Edible Food Coatings

Biopolymers have emerged as a viable option for food coating applications due to their biodegradability, non-toxicity, and film-forming properties [160,161,162]. Biopolymers used in food coatings are structurally composed of proteins, polysaccharides, and lipids, each with distinct qualities that contribute to their efficiency. Because of the structural diversity of biopolymers, specific food coating applications may be tailored, enhancing food safety and increasing shelf life in an environmentally responsible way. Casein, whey, and soy protein are examples of proteins with molecular weights that range from 14,000 to 600,000 Daltons. Their cohesive and strong coatings have high oxygen barrier properties but poor moisture barrier capabilities. The molecular weight of polysaccharides, which range from 50,000 to over 10 million Daltons, includes chitosan, starch, alginate, and derivatives of cellulose. These biopolymers function well as gas and scent barriers, and chitosan has antibacterial qualities as well. Their ability to withstand moisture differs greatly. In general, lipids with molecular weights of a few hundred Daltons or less include waxes and fatty acids. They are frequently used with polysaccharides or proteins to enhance the moisture barrier qualities of composite films. Although they are less successful at keeping moisture out, proteins like casein, whey, and soy are recognized for their ability to build robust, cohesive films with exceptional mechanical qualities. Polysaccharides such as chitosan, starch, alginate, and cellulose derivatives are appreciated for their capacity to create gels and films that effectively block gasses and aromatic chemicals, while moisture resistance varies. For instance, chitosan has both strong film-forming capabilities and antibacterial qualities. In biopolymer coatings, lipids, including fatty acids, waxes, and emulsifiers, are frequently added to improve moisture resistance. These composite films have a wide range of uses in food coating, including long-term preservation and increased shelf life [163,164].

## 10. Conclusions

In conclusion, biopolymers are a viable alternative for food coating applications due to their biodegradability, renewability, and ability to improve food quality and safety. They provide a sustainable alternative to synthetic coatings, decreasing environmental impact while addressing customers’ desire for healthier and more natural food items. Biopolymers are set to revolutionize food coating applications due to their eco-friendliness and versatility. Natural polymers, originating from microbes and plants, can decompose naturally, which helps to alleviate the environmental issues related to conventional synthetic coatings. Their capacity to construct efficient barriers against moisture, oxygen, and pollutants increases shelf life and food safety while also addressing major challenges in food preservation and waste reduction. To guarantee their cost-effectiveness, enhance their applicability to a wider range of food kinds and processing circumstances, and improve their features, more study is necessary. Biopolymers have the potential to totally transform food packaging and preservation practices in the future with further research and development. Notwithstanding these benefits, problems with scalability and production costs still exist. In order to outperform traditional plastics in terms of cost and competitiveness, modern production processes are required. In addition, ongoing research must focus on improving biopolymer performance under a range of environmental conditions in order to preserve reliability across the food supply chain. Biopolymers represent a significant advancement in sustainable packaging and preservation solutions for the food industry. Continued investment in research and development is projected to realize its full potential, propelling the adoption of biopolymer coatings as a standard for environmentally responsible food packaging and ensuring a more sustainable future for our food systems.

## Figures and Tables

**Table 1:** Recent trends on bioactive materials for the edible food coating applications.

Scheme	Edible Coating Material	Technique	Observations and Shelf-Life Effectiveness	Food Product	Ref.
1	Alginate-oleic acid	Spreading	Antiviral activity	Strawberries and raspberries	[151]
2	Essential oil of turmeric/ginger/clove	Heating and Drying	Coated tomatoes and Amla remained fresh for a longer time as compared to non-coated samples	Tomatoes and Amla	[152]
3	Chitosan	Dipping	The color change was delayed, and Alternaria alternata growth was inhibited	Figs	[153]
4	Alginate	Blending	Inhibition of the growth of the fungal pathogen Colletotrichumgloeosporioides at 10 °C storage	Capsicum	[154]
5	Chitosan/Carboxy methyl cellulose	Nano-emulsified coatings	Antibacterial protection (~5 log reduction) and extended storability (13 days)	Fresh-cut melons	[155]
6	Whey protein	Spreading	Inhibition action against *E. Coli*/*L. Monocytogenes*	Beef	[156]
7	Alginate	Spreading	Improved microbial activity	Abalone	[157]
8	Corn starch	Spreading	Enhanced activity towards Bacillus cereus and Aspergillus Niger	Sweet meat/Doda Burfi	[158]
9	Zein nanofibers	Coating	Outstanding antibacterial activity against *S. Aureus* and L/Mmonocytogenes, over ~28 days	Cheese	[159]
